# Identification and Analysis of Potential Autophagy-Related Biomarkers in Endometriosis by WGCNA

**DOI:** 10.3389/fmolb.2021.743012

**Published:** 2021-11-01

**Authors:** Jing Wang, Shanshan Cong, Han Wu, Yanan He, Xiaoli Liu, Liyuan Sun, Xibo Zhao, Guangmei Zhang

**Affiliations:** ^1^ Department of Gynecology, The First Affiliated Hospital of Harbin Medical University, Harbin, China; ^2^ Department of Gynecology, The Red Cross Center Hospital of Harbin, Harbin, China

**Keywords:** endometriosis, autophagy-related genes (ATGs), weighted gene co-expression network analysis (WGCNA), EZH2, RND3

## Abstract

**Background:** Endometriosis is a serious gynecological disorder characterized by debilitating pain, infertility and the establishment of innervated endometriosis lesions outside the uterus. Early detection and accurate diagnosis are pivotal in endometriosis. The work screened autophagy-related genes (ATGs) as potential biomarkers to reveal new molecular subgroups for the early diagnosis of endometriosis.

**Materials and Methods:** The gene lists of ATGs from five databases were integrated. Then, weighted gene co-expression network analysis (WGCNA) was used to map the genes to the gene profile of endometriosis samples in GSE51981 to obtain functional modules. GO and KEGG analyses were performed on the ATGs from the key modules. Differentially expressed ATGs were identified by the limma R package and further validated in the external datasets of GSE7305 and GSE135485. The DESeq2 R package was utilized to establish multifactorial network. Subsequently, one-way analysis of variance (ANOVA) was performed to identify new molecular subgroups. Real-time quantitative polymerase chain reaction (RT-qPCR) and Western blotting were used to confirm the differential expression of hub ATGs, and the receiver operating characteristic (ROC) curve analysis and Spearman correlation analysis were applied to assess the diagnostic value of hub ATGs in 40 clinical samples and human primary endometrial stromal cells (ESCs).

**Results:** We screened 4 key modules and 12 hub ATGs and found the key genes to be strongly correlated with endometriosis. The pathways of ATGs were mainly enriched in autophagy, apoptosis, ubiquitin-protein ligase binding, and MAPK signaling pathway. The expression levels of EZH2 (Enhancer of Zeste homolog 2) and RND3 (also known as RhoE) had statistically significant changes with higher values in the endometriosis group compared with the controls, both in the tissue samples and primary ESCs. Besides, they also showed higher specificity and sensitivity by the receiver operating characteristic analysis and Spearman correlation analysis for the diagnosis of endometriosis. The TF-mRNA-miRNA-lncRNA multifactorial network was successfully constructed. Four new molecular subgroups were identified, and we preliminarily showed the ability of IQCG to independently differentiate subgroups.

**Conclusion:** EZH2 and RND3 could be candidate biomarkers for endometriosis, which would contribute to the early diagnosis and intervention in endometriosis.

## Introduction

Endometriosis is a chronic, inflammatory, oestrogen-dependent disease, in which endometrial stromal cells and glands are implanted outside the uterine cavity (Bulun et al., 2019). It affects approximately 10% of the women of reproductive-age and leads to a marked reduction in the patients’ quality of life (QoL) (Vallvé-Juanico et al., 2019). So far, no consistent theory regarding the etiological factors of endometriosis has been established. In addition, it is hard to be diagnosed at early stages, and the available clinical methods are limited.

Recent studies have indicated that autophagy plays an integral role in the physiological and pathophysiological mechanisms associated with the endometria, including the menstrual cycle, decidualization and vascular remodeling of normal pregnancy, as well as endometrium-related diseases, such as endometriosis, infertility or subfertility, and endometrial carcinoma (Yang et al., 2017; Yang et al., 2019b). In consistence with the changes in autophagy levels between ectopic and eutopic endometria in endometriosis, an aberrant expression of autophagy-related genes has also been observed (Luo et al., 2018; Mei et al., 2018). Many factors have been shown to contribute to abnormal autophagy in endometrial tissues, including estrogen, certain drugs, oxidative stress, and hypoxia (Rižner, 2009; Zhao et al., 2018). Besides, autophagy has been connected with the recurrence and development of endometriosis (Ma et al., 2019; He et al., 2020). Large-scale screening and bioinformatics tools revealed the abnormal expression of ATGs to be closely related to the initiation, development, early diagnosis and poor prognosis of tumors, such as gastric carcinoma, prostate cancer, and lung cancer (Yepes et al., 2016; Huang et al., 2017). However, the connection between hub ATGs and endometriosis still needs to be exploited.

Weighted gene co-expression network analysis (WGCNA) represents one of the most important and widely used methods of systems bioinformatic (Langfelder and Horvath, 2008), which is used to describe the correlation patterns among genes via microarray specimen (Langfelder and Horvath, 2012). It can be executed through an R package to group the genes into modules according to the gene co-expression similarities across the samples. The WGCNA method can not only retrieve a cluster of genes with similar biological functions, but it may connect modules to external clinical features. As a result, relevant functional networks can be used to identify specific biomarkers and new molecules (Liu et al., 2017; Pascut et al., 2020).

In this work, we present an innovative investigation of the association between ATGs and the endometriosis clinical traits. EZH2 and RND3 were proven to be candidate diagnostic biomarkers and validated by external datasets and experiments. Four new molecular subgroups were identified, and a multifactorial network was constructed. The insights from this work lay a theoretical foundation for the early diagnosis and prompt treatment of endometriosis.

## Materials and Methods

### Data Preparation

We used the Gene Expression Omnibus (GEO, https://ncbi.nlm.mih.gov/geo/), which is a public functional genomics database with various types of gene profiles in various diseases. We retrieved multiple GEO series, including mRNA (GSE51981, GSE135485, GSE7305), miRNA (GSE105765), and lncRNA (GSE105764) datasets. Details of the retrieved data are listed in Table 1. Then, we integrated the ATGs derived from HADb (http://www.autophagy.lu/index.html), AUTOPHAGY DATABASE (http://www.tanpaku.org/autophagy/index.html), HAMdb (http://hamdb.scbdd.com/), GSEA (https://www.gsea-msigdb.org/gsea/index.jsp) and NCBI (https://www.ncbi.nlm.nih.gov/). A total of 2,209 genes were extracted, among which 1928 autophagy-related genes were expressed in the GSE51981 dataset (See Supplemental Table S1).

**TABLE 1:** Sample information of endometriosis in database.

	Sample	Control	Endometriosis	All
mRNA	GSE51981	71	77	148
mRNA	GSE135485	4	54	58
mRNA	GSE7305	10	10	20
miRNA	GSE105765	8	8	16
lncRNA	GSE105764	8	8	16

### Weighted Gene Co-Expression Network Analysis

We applied the WGCNA method to construct the gene co-expression network and identify the functional modules (Langfelder and Horvath, 2008). Hierarchical clustering analysis was performed using the R software package gplots and the sample outliers were identified. The WGCNA algorithm was used to calculate the ATGs correlation among the samples. Then the appropriate soft threshold power was performed, and the standard scale free network was established. The models or networks that were obtained using WGCNA were relevant to the external sample features. Next, the correlation functional networks were used to identify tissue-specific markers. The modules were identified using a dynamic tree-cut strategy by clustering the genes hierarchically. Next, WGCNA was used to construct the gene co-expression modules and extract the gene information in each module. A total of 1928 autophagy-related genes (ATGs) were mapped to the expression profile of the GSE51981 samples, and key functional modules with highly correlated genes were identified.

### Enrichment Analysis of Key Modules and Identification of Hub ATGs

According to the obtained gene significance scores, the numbers of genes in key modules were arranged in sequence from high to low. Next, functional enrichment analysis was performed to analyze, identify, and interpret diverse biological functions based on the Kyoto Encyclopedia of Genes and Genomes (KEGG) pathway analysis and Gene Ontology (GO) annotation analysis in the functionally related modules. The data were visualized and analyzed using the ClusterProfiler and ggplot2 R packages (*pvalueCutoff* = 0.05).

Hub genes represent the top-ranked genes in the inter-modular connectivity, which are centrally located in their respective module and can reflect its characteristics. Compared with the other genes in the network, the hub genes of key modules have a greater biological significance. Using the limma R package, differential expression analyses were performed between the endometriosis group and control group on the GSE51981 dataset (*p* < 0.05) (Ritchie et al., 2015). WGCNA was used to identify gene-gene interactions in each notable module, and the hub ATGs were then predicted using CytoHubba (Degree method, Rank <1) (Chin et al., 2014). The above-mentioned data were merged, and the overlaps were calculated. In order to further confirm the significance and accuracy of the screening genes, the expression of the resulting hub ATGs were validated using two external datasets: GSE7305 (ten control samples, ten endometriosis samples) and GSE135485 (four control samples, fifty-four endometriosis samples).

### New Molecular Subgroups and Multifactorial Network

Based on the expression of hub ATGs in 77 samples from GSE51981, we performed a clustering analysis of the dataset using the ConsensusClusterPlus R package to recognize new molecular subgroups (Wilkerson and Hayes, 2010). As a result, we obtained the item-consensus results and cluster consensus. One-way analysis of variance (ANOVA) was used to determine whether the expression of the hub ATGs significantly varied among the molecular subgroups (ANOVA, *p* < 0.05).

We retrieved the miRNA and mRNA interactions from the miRNA 3.0 database (http://mirwalk.umm.uni-heidelberg.de/), under the condition that the interaction relationships were predicted from all four databases (miRNA, TargetSan, miRDB, miRTarBase). We retrieved the interaction relationship between miRNA and lncRNA from the StarBase v3.0 database (http://starbase.sysu.edu.cn) and the relationship between the TF and mRNA from the TRRUST v2 database (http://www.grnpedia.org/trrust/). Finally, we used the DESeq2 R package (Love et al., 2014) to analyze the differential expression of miRNA and lncRNA.

### Collection of Tissue Samples and Isolation of Human Primary ESCs

Twenty female patients aged 29–46 years who underwent surgery for endometriosis (endometriosis group) and twenty female patients aged 26–43 years who underwent surgery for idiopathic infertility (control group) in the First Affiliated Hospital of Harbin Medical University (Harbin, PR China) were enrolled in the study. The ectopic endometrium tissues were obtained from the endometriosis group and the normal endometrial tissues were obtained from the control group. Detailed clinical characteristics of all samples were summarized in Table 2. All the patients provided an informed consent form before recruitment and the research protocol was approved by the Ethics Commission of Harbin Medical University (202105).

**TABLE 2:** Clinical Characteristic of tissue samples.

Characteristic	Endometriosis (*n* = 20)	Control (*n* = 20)
Age	36.8 ± 4.6	33.5 ± 3.5
Main Diagnose	Ovarian Endometriosis	Idiopathic Infertility
phases of the menstrual cycle		
Proliferation phase	14	15
Secretory phase	6	5
Pretherapy clinical staging		
Stage I-II	12	NA
Stage III-IV	8	NA
Distribution of Endometriosis		
Ovarian Endometriosis	13	NA
Dysmenorrhea	4	NA
Peritoneal Lesion	3	NA
Pelvic adhesion		
President	13	9
Absent	7	11
DIE status		
With DIE lesions	5	NA
Without DIE lesions	15	NA
CA125 (mean ± SD)		
Stage I-II	85.6 ±15.3	NA
Stage III-IV	108.4 ± 16.7	NA

NA: not applicable.

DIE: deep infiltrating endometriosis.

Primary normal endometrial stromal cells (NESCs) and ectopic endometrial stromal cells (EESCs) were isolated from fresh tissues according to the previously published methods (Gargett et al., 2009). Tissues were transported to the laboratory in an ice-cold medium (DMEM/F-12 1:1) within 2 h, washed twice with sterile PBS to remove the blood and then minced into small pieces and digested in type IV collagenase (Life Technologies) at 37°C for 60–90 min. The cell suspension was obtained after filtering through a 70-μM sieve to remove debris and glandular epithelial cells. The ESCs suspension was collected and centrifuged at 800 rpm for 5 min at room temperature. The isolated cells were maintained in the medium (DMEM/F-12 1:1) containing 10% fetal bovine serum and 1% antibiotic penicillin-streptomycin (Gibco) at 37°C and 5% CO2. The culture medium was changed every 3 days. The purity of ESCs was detected by separate immunofluorescence for the epithelial marker Cytokeratin 7 (Abcam) and stromal marker Vimentin (Abcam). Only the study that measured Vimentin positive cells at more than 95% were included.

### Real-Time Quantitative Polymerase Chain Reaction

Total RNA was extracted from the patients’ specimens and human primary ESCs using Trizol reagent (Invitrogen, United States), and it was reverse transcribed to cDNA using a reverse transcription kit (TaKaRa, Japan) following the provided instructions. Quantitative PCR was performed using SybGreen by the real-time PCR detection system (Yang et al., 2019a). A total volume of 20 ul was subjected to the PCR process: 95°C for 30 sec, 40 cycles of 95°C for 5 sec, and 60°C for 30 sec. Then, relative mRNA levels were calculated using the comparative CT method (2^−△△^Ct) (Zhou et al., 2018). Primer sequences used in the study are described in Table 3.

**TABLE 3:** Primer sequences for RT-qPCR.

	Forward	Reverse
EZH2	GGA​CCA​CAG​TGT​TAC​CAG​CAT	GTG​GGG​TCT​TTA​TCC​GCT​CAG
IQCG	TCC​CTT​CCA​GAT​GTG​CTG​AG	ACG​GGC​ATG​ATG​TAG​TTC​AGA
RCAN1	GCG​TGG​TGG​TCC​ATG​TAT​GT	TGA​GGT​GGA​TCG​GCG​TGT​A
RND3	GCT​CCA​TGT​CTT​CGC​CAA​G	AAA​ACT​GGC​CGT​GTA​ATT​CTC​A
PPP2R5E	CAG​AGG​CTG​TTT​GAC​AGA​GCA	TCA​CTA​GGA​GGG​AGA​GTT​CTG​A
EPHA5	GTG​ACC​GAT​GAA​CCT​CCC​AAA	CCA​GGT​CTG​CAC​ACT​TGA​CAG

### Western Blotting Analysis

Total protein was extracted from both the tissues and cells following the instructions of the RIPA lysis buffer (Sigma, United States). Next, total protein concentration was measured using the BCA assay (Beyotime, China). Total proteins (30 ug) were separated on a 4–12% polyacrylamide gel by SDS-PAGE and electrophoretically transferred onto PVDF membranes, subsequently sealed with 5% BSA. The membranes were incubated with diluted primary antibodies to EZH2 (1:2,000 dilution, Abcam), RND3 (1:1,000 dilution, Abcam), IQCG (1:1,000 dilution, Abcam) overnight at 4 °C. The membrane was then further incubated with HRP-labelled secondary antibodies (Jackson, United States) at room temperature for 1 h. Finally, protein bands were analyzed and quantified using the ImageJ software.

### Statistical Analysis

All statistical analyses were established using GraphPad Prism 8.0 and SPSS v20.0. The statistical significance of pairwise differences between the groups was indicated by an unpaired *t*-test, and a *p* ≤ 0.05 was considered to be statistically significant. The receiver operating characteristic (ROC) curve analysis and Spearman correlation analysis were used to evaluate the diagnostic value of the hub ATGs and the correlation of the hub ATGs with cancer antigen 125 (CA125).

## Results

### Selection of Key Modules

**FIGURE 1:**
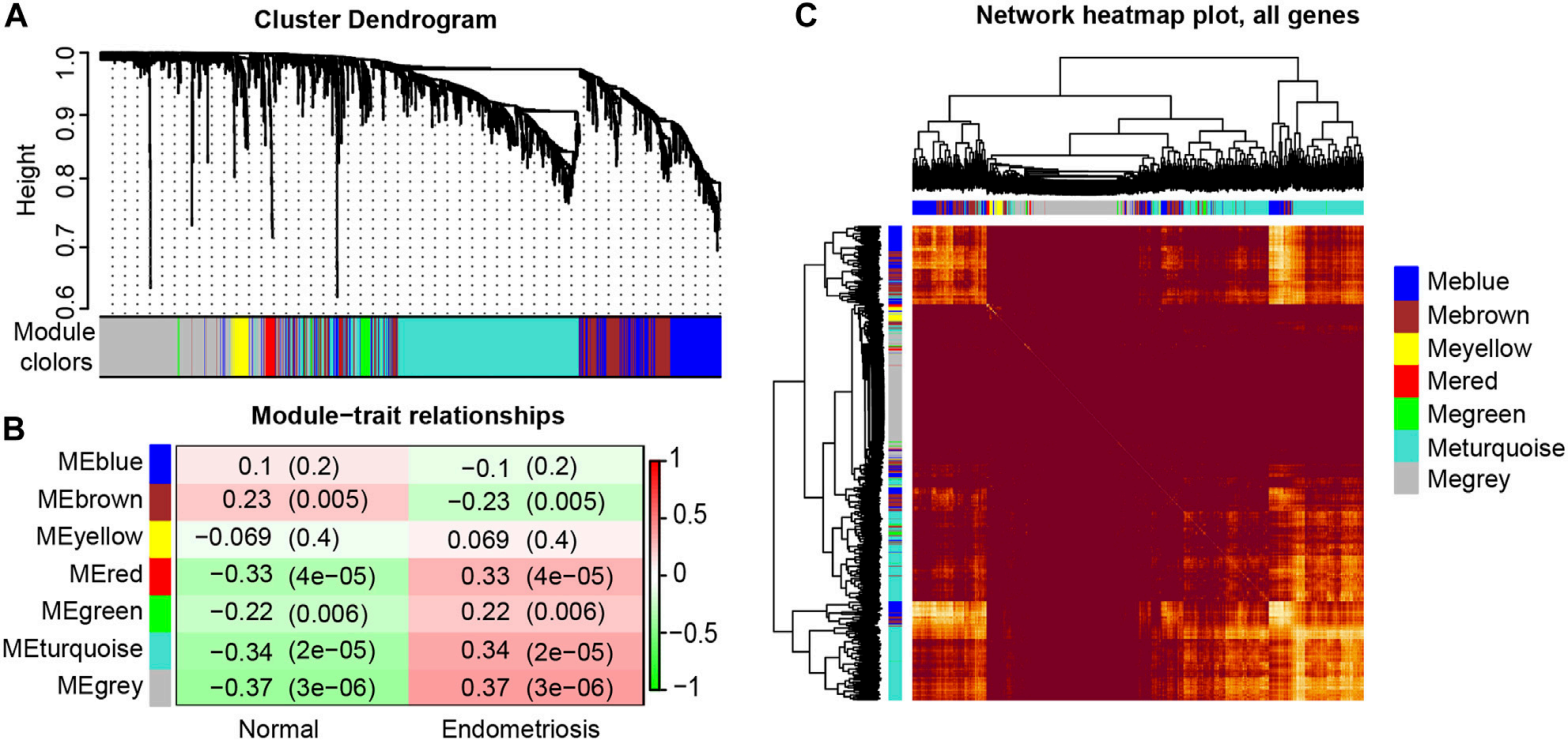
Identification and characterization of endometriosis-associated autophagy-related genes (ATGs) key modules. Hierarchical cluster tree showing co-expression modules based on WGCNA **(A)**. Correlation between seven key modules and clinical traits **(B)**. Co-expression heat map and correlations for ATGs in models **(C)**.

In our study, seven modules (indicated by the colors of Blue, Brown, Yellow, Red, Green, Turquoise and Grey) were identified according to the average linkage hierarchical clustering (Figure 1A, see Supplemental Table S2 for the number of genes in each module). The analysis of the relationship between the gene co-expression modules and clinical traits resulted in several findings. The genes in the Brown module were positively correlated with the clinical traits of the control group and negatively correlated with those of the endometriosis group. However, the Red, Green, Turquoise, and Grey modules were negatively correlated with the clinical features of the control group and positively related to those of the endometriosis group (Figure 1B). At last, four key modules (the Brown, Turquoise, Green, and Red modules) were identified. The heat map based on the co-expressed genes showed that ATGs in the same model tended to have a high biological relevance (Figure 1C).

### GO and KEGG Enrichment Analysis

**FIGURE 2:**
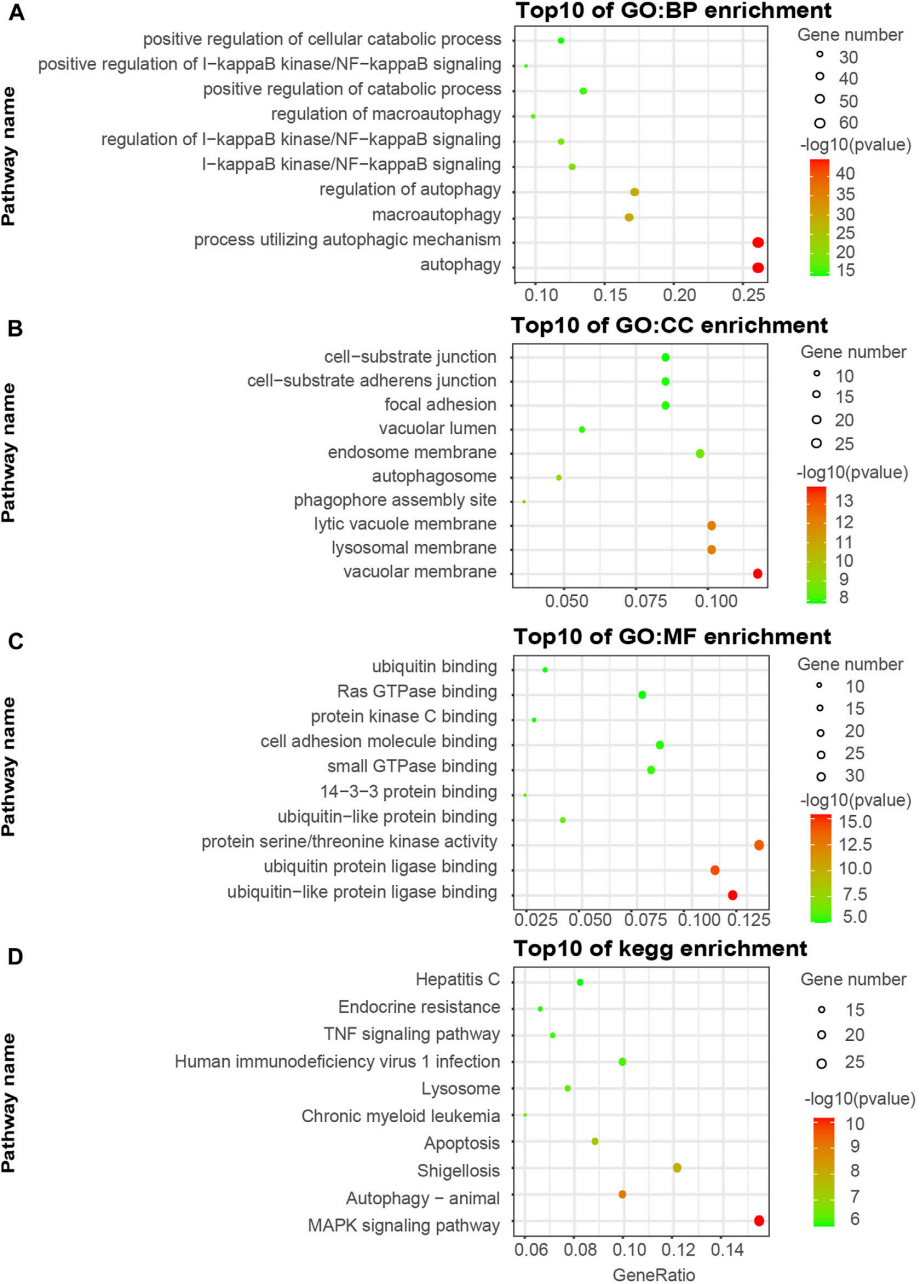
Gene ontology (GO) and KEGG pathway enrichment analyses of ATGs in Brown **(A–D)** modules.

**FIGURE 3:**
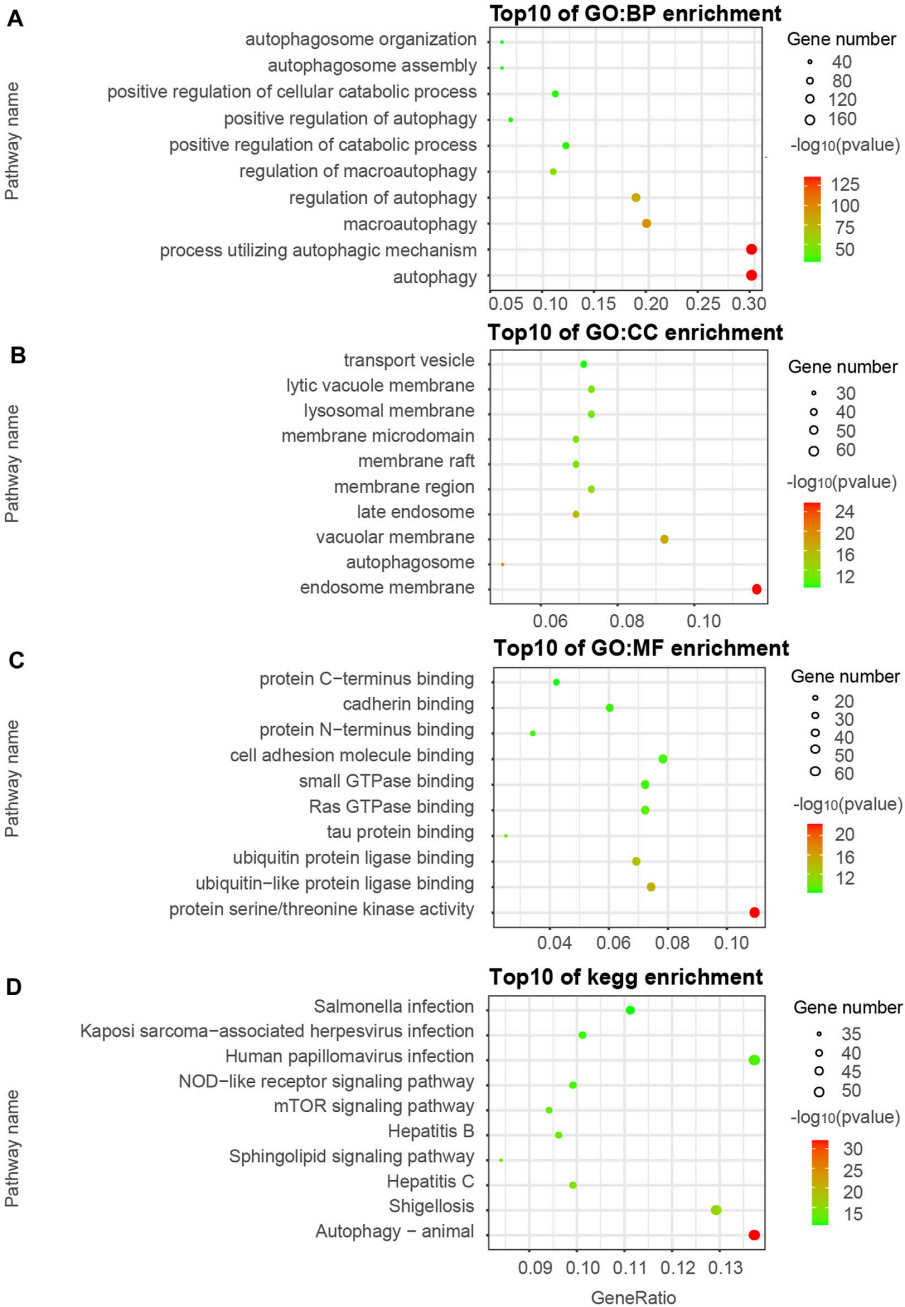
Gene ontology (GO) and KEGG pathway enrichment analyses of ATGs in Turquoise **(A–D)** modules.

We visualized the top ten pathways resulting from the GO and KEGG pathway analysis. The analysis resulted in 254 ATGs in the Brown model, as shown in Figures 2A–D. They mainly focused on the signaling pathways associated with the components of autophagy, apoptosis, ubiquitin-protein ligase binding, and MAPK signaling pathway. In addition, the analysis resulted in 589 ATGs in the Turquoise module, as shown in Figures 3A–D, which focused on autophagy and protein serine/threonine kinase activity pathway. In the Green model, 35 ATGs resulted from the analysis, as shown in Supplemental Figures 1A–C, which were mainly related to the inflammatory mediator regulation of TRP channels. Finally, in the Red module, the analysis resulted in 28 ATGs, as shown in Supplemental Figures 1D–F, which were mainly enriched in cell cycle.

### Identification and Validation of 12 Hub ATGs in External Datasets

**FIGURE 4:**
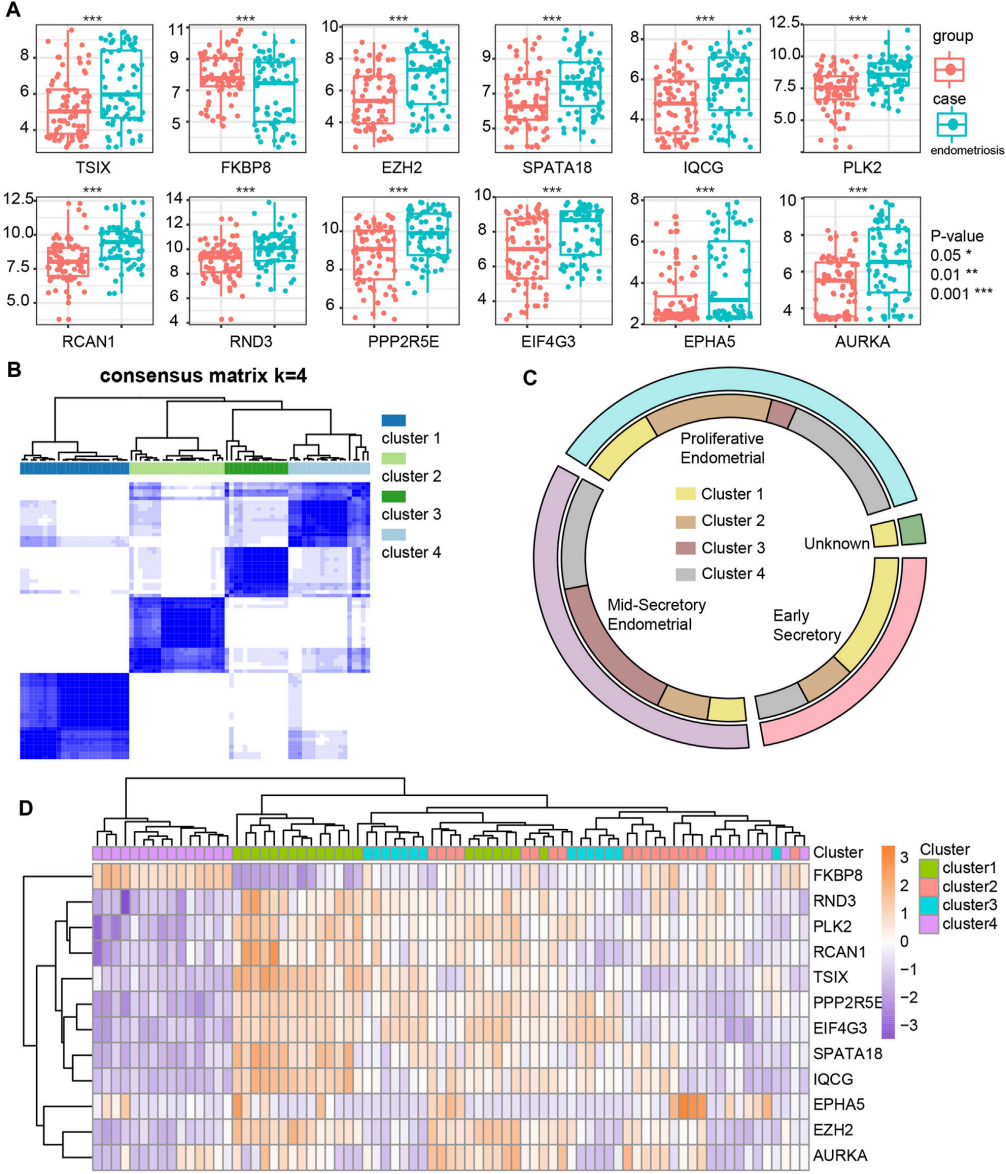
Identification and differential expression of hub ATGs in GSE51981 **(A)**. Four new molecular subgroups were performed by consensus clustering analysis in endometriosis **(B)**. The distribution of the four new molecular subgroups in different periods of endometrium **(C)**. The expression of the 12 hub ATGs in four new molecular subgroups **(D)**.

We screened the ATGs of key modules and retained co-expressed gene pairs. Then, hub genes obtained by the cytoHubba were overlapped with the differentially expressed ATGs in GSE51981 (Supplemental Table S3). As a result, we obtained 12 hub ATGs (TSIX, FKBP8, EZH2, SPATA18, IQCG, PLK2, RCAN1, RND3, PPP2R5E, EIF4G3, EPHA5, AURKA), the typical box plot is shown in Figure 4A. Notably, the expression level of each hub ATG was significantly higher in the endometriosis group compared with the control group (***p < 0.001). Then, we validated the 12 hub ATGs in the two external datasets of GSE7305 and GSE135485. The comparison of the expression levels of the hub ATGs between the endometriosis group and control group is shown in Supplemental Figures 2A,B, normalized by log2 (*n* + 1). These validation results were also roughly consistent with the results obtained from GSE51981.

### New Molecular Subgroups in Endometriosis

**FIGURE 5:**
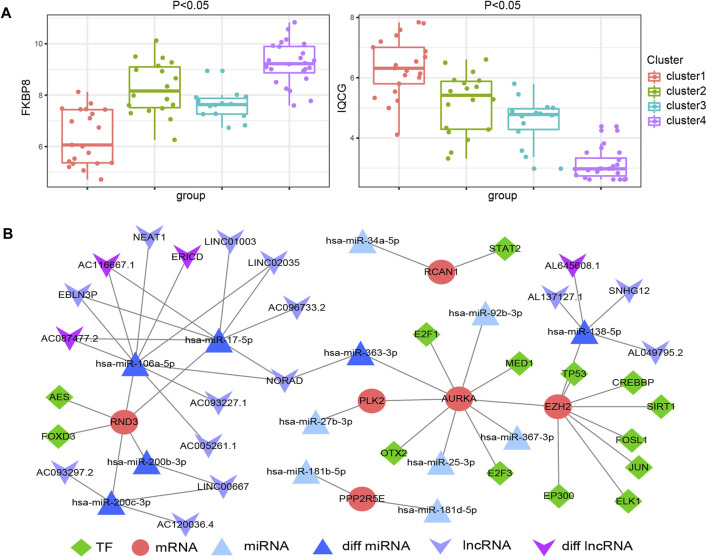
Differential expression of FKBP8 and IQCG in the four new subgroups of endometriosis **(A)**. Construction of the multifactorial network in endometriosis **(B)**.

We mapped 1928 ATGs to the gene expression profile of endometriosis GSE51981, which was performed by consensus clustering analysis. Endometriosis samples were appropriately classified into four subgroups, as shown in Figure 4B (At K = 4, cluster heat map). Our results showed 21, 18, 14, and 24 samples in cluster 1, cluster 2, cluster 3, and cluster 4, respectively. We also analyzed the expression of the 12 hub ATGs in the different clusters (Figure 4D). The distribution of the four new molecular subgroups in different periods of the uterus endometrium is illustrated by a Circos plot in Figure 4C, in this figure, four subgroups are shown in the inner circle, corresponding to different periods of endometrium samples in the outer circle. Statistical comparisons of the 12 hub ATGs using one-way ANOVA resulted in four significantly different ATGs (*p* < 0.05). Then, we further investigated the expression levels of two representative genes (FKBP8, IQCG) in different clusters (Supplemental Table S4). The results showed that both FKBP8 and IQCG were obviously differentially expressed among the four subgroups (Figure 5A). The above-mentioned results suggested that FKBP8 and IQCG could potentially serve as independent factors to discriminate between different subgroups.

### A Multifactorial Network Construction

A total of 27 miRNA-hub ATGs were acquired from the miRwalk 3.0 database according to the interaction relationship in 3′ UTR, 5’ UTR and CDS regions (Supplemental Table S5). Besides, 26 miRNA-lncRNA interaction relationships were obtained based on the StarBase v3.0 database (Supplemental Table S6), and 28 TF-mRNA interaction relationships were retrieved from the TRRUST v2 database (Supplemental Table S7). The analysis of variance on GSE105765 and GSE105764 using the R package DESeq2 resulted in six lncRNA and four miRNA that were overlapped between the above-mentioned analysis results and network relationships. Thus, we successfully constructed the TF-mRNA-miRNA-lncRNA multifactorial network, as shown in Figure 5B.

### ROC Analysis and Spearman Correlation Analysis

**FIGURE 6:**
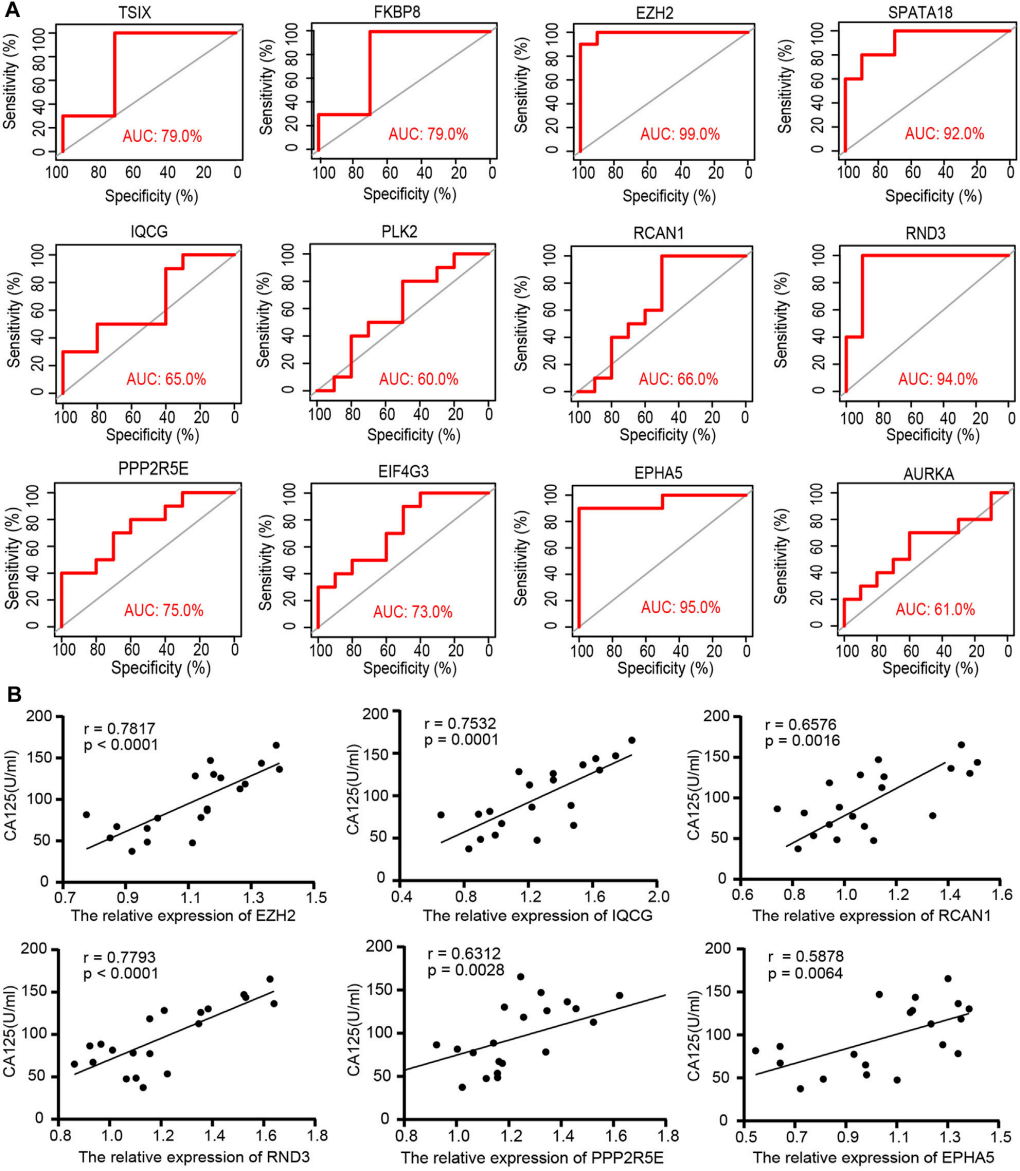
The specificity and sensitivity of the 12 hub ATGs for the diagnosis of endometriosis by the receiver operating characteristic (ROC) analysis **(A)**. The relative expression of the 6 hub ATGs in tissues were positively correlated with Ca125 by Spearman correlation analysis **(B)**.

The ROC analysis was employed to evaluate the specificity and sensitivity of the 12 hub ATGs for the diagnosis of endometriosis. The accuracy of the score was assessed by calculating the ROC curve and analyzing the area under the receiver operating characteristic curve (AUC) following previous strategies (Yamamoto et al., 2013; Roy et al., 2021). As shown in Figure 6A, the area under the curve (AUC) values of all 12 hub ATGs were more than 60% in GSE7305. Among these, the AUC values of EZH2, SPATA18, RND3, and EPHA5 exceeded 90%, which means that the hub genes had high sensitivity and specificity. Cancer antigen 125 (CA125) is generally present in normal ovarian epithelia, endometrium and decidua (Liu et al., 2021), and its normal level is <35 U/ml. CA125 is often elevated in benign conditions such as endometriosis and ovarian cysts (Whitwell et al., 2020). It has been a traditional marker for ovarian tumor screening. Spearman correlation analysis depicted that the relative expression levels of EZH2 and RND3 were positively correlated with CA125 of the corresponding endometriosis samples, (r = 0.7817, *p* < 0.0001) and (r = 0.7793, *p* < 0.0001), respectively, (Figure 6B). These results indicated that EZH2 and RND3 could serve as novel biomarkers for endometriosis.

### RT-qPCR and Western Blotting Results of the Hub ATGs

**FIGURE 7:**
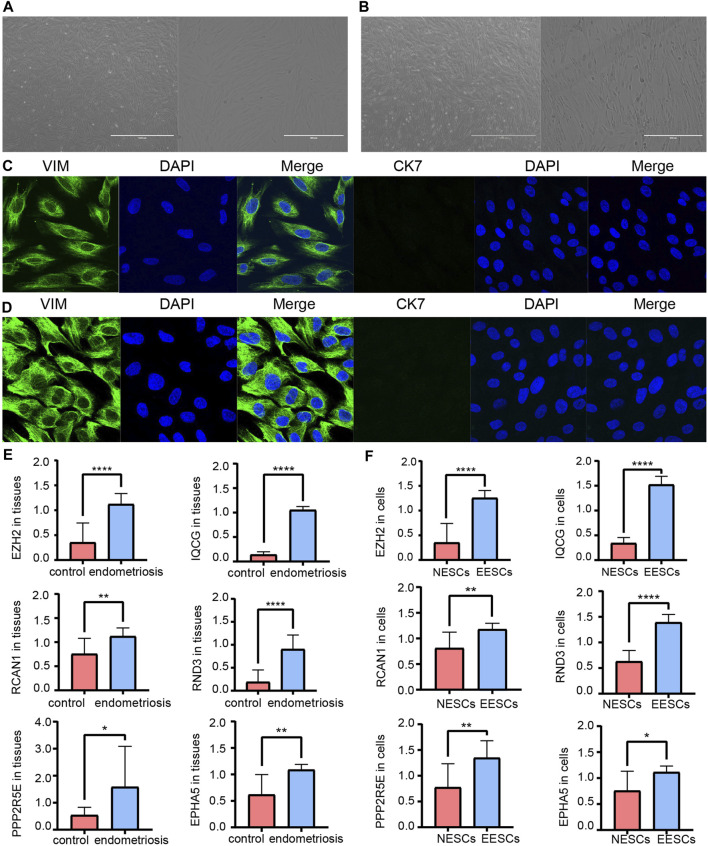
Morphology of primary NESCs aggregates **(A)**. Morphology of primary EESCs aggregates **(B)**. Identification of primary NESCs **(C)**. Identification of primary EESCs **(D)**. The differences in the expression levels of EZH2, IQCG, RCAN1, RND3, PPP2R5E, and EPHA5 by RT-qPCR between the endometriosis tissues and the control tissues **(E)**, those between NESCs and EESCs **(F)**.

**FIGURE 8:**
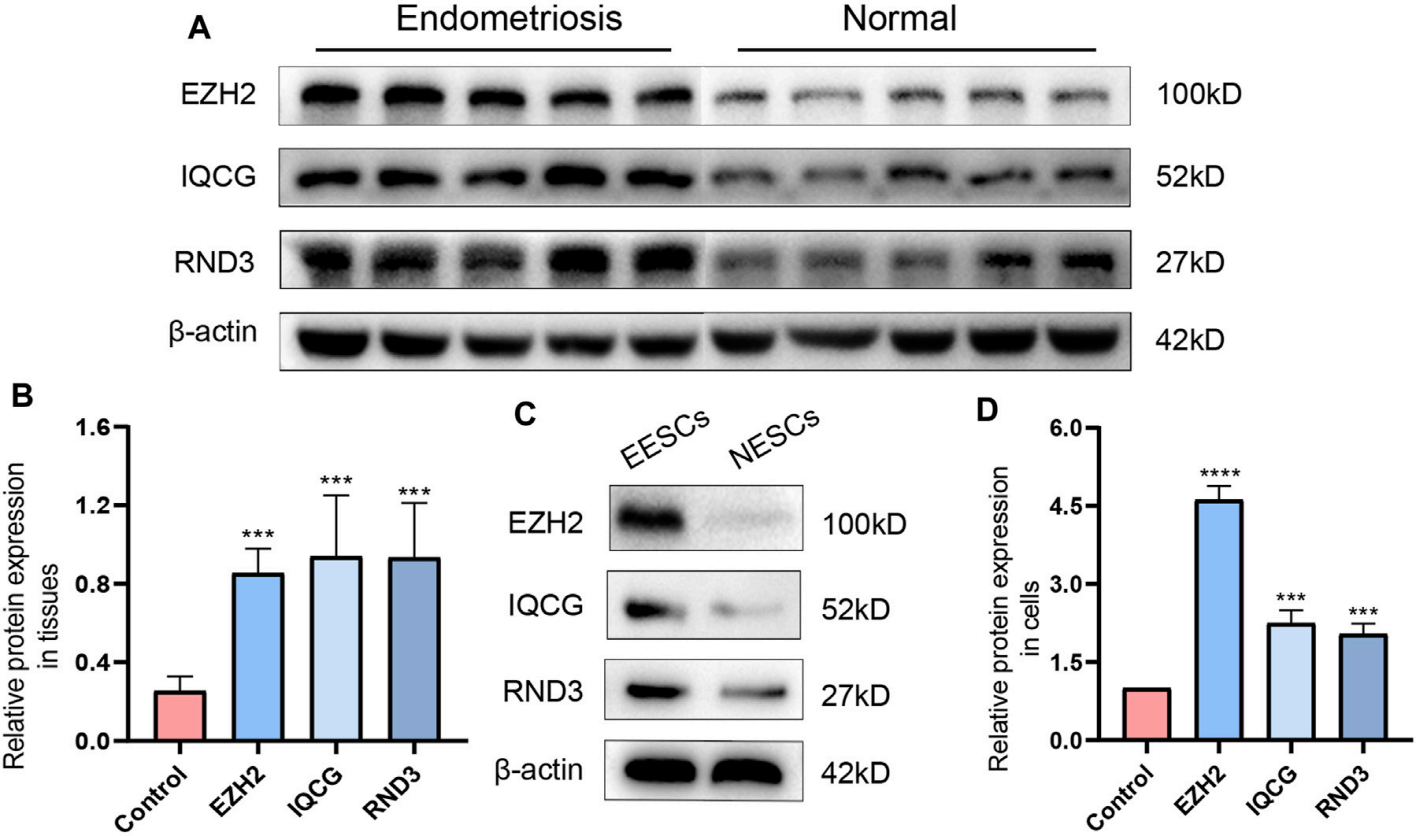
The differences in the expression levels and quantitative analysis of EZH2, IQCG, and RND3 by Western blotting between the endometriosis tissues and the control tissues **(A,B)**, those between NESCs and EESCs **(C,D)**.

Measuring the expression of 12 hub ATGs using RT-qPCR showed that the expression levels of EZH2, IQCG, RCAN1, RND3, PPP2R5E, and EPHA5 were higher in the ectopic endometrial tissues compared with the normal endometrial tissues (*p* < 0.05), which was consistent with the expression pattern of these six genes in multiple databases (Figure 7E). Among them, the differences in the expression levels of EZH2, IQCG, and RND3 between the endometriosis group and control group were the most statistically significant (*p* < 0.001). Then, primary NESCs and EESCs were successfully isolated from the fresh normal endometrial tissues and ectopic endometrial tissues (Figures 7A,B). The purity of NESCs and EESCs was identified using cellular immunofluorescence separately for the epithelial marker Cytokeratin 7 (Abcam) and stromal marker Vimentin (Abcam) (Figures 7C,D). We only selected Vimentin cells that were positive for more than 95% from P3-P5 for further research. The RT-qPCR results showed that the levels of EZH2, IQCG, and RND3 in EESCs were significantly higher than those in NESCs (Figure 7F). This further verified the above conclusion. In view of above results, protein expression of EZH2, RND3, and IQCG were subsequently measured in endometriosis cells and tissues by Western blotting (Figures 8A–D), which demonstrated similar results to those of the mRNA level.

## Discussion

In the study, the GO and KEGG enrichment analyses indicated that the ATGs were predominately associated with autophagy-related functions and pathways, such as the processes utilizing autophagic mechanism, autophagosome, apoptosis, cell cycle, and MAPK signaling pathway. The two functions of autophagy and apoptosis are interconnected with each other (Guo et al., 2016). In general, the most common conclusion is that basal macroautophagy was detectable at all cell cycle stages. The cell cycle regulators might directly regulate macroautophagy to accelerate the mobilization and activity of DNA damage/repair complex, especially in response to DNA damage (Zheng et al., 2019). In addition, it has been shown that under anoxic conditions, inhibiting autophagy by beclin1 siRNA promoted apoptosis in human endometrial stromal cells. MTOR inhibition was involved in autophagy induction to promote endometriotic cell apoptosis (Choi et al., 2014; Liu et al., 2019). In our study, GO and KEGG analysis showed that ATGs might potentially play a remarkable role in regulating autophagy and apoptosis in endometriosis. Integrin-mediated intracellular ERK/MAPK acting downstream and MAPK activation were correlated with endometriosis, which were both shown to be involved in autophagy and apoptosis, gene expression, cell division, movement, and survival (Uimari et al., 2017). Besides, a previous study revealed that iron modulated cell autophagy in a Ras and MAPK-dependent manner in ovarian cancer cells (Bauckman et al., 2013). Our findings further confirmed the involvement of ATGs in the regulation of the MAPK signaling pathways in endometriosis. This shows the importance and essentiality of conducting research on ATGs in endometrial-related diseases. These findings have laid the foundation for the screening of new candidate biomarkers and the early diagnosis and treatment of endometriosis.

Molecular subtypes have been defined in autoimmune diseases and various cancers types. Therefore, determining the molecular subtypes of endometriosis is crucial for personalized treatment. The relationships between molecular subtypes and clinical characteristics need to be further evaluated. We performed clustering analysis of the samples in GSE51918 using ConsensusClusterPlus and further classified the endometriosis cases into 4 new molecular subgroups. This revealed that ATGs from different subgroups exhibited different clinical characteristics. Starting from cluster 1, the ATGs (EIF4G3, SPATA18, IQCG) were dominant in the early secretory phase of endometrial tissues. In cluster 3, the ATGs (mainly PPP2R5E and EIF4G3) were characterized by being dominant in the middle-secretory phase of endometrial tissues. As for cluster 2 and cluster 4, they were mainly enriched in the endometrial proliferative phase. EPHA5 is highly expressed in cluster 2 but barely expressed in the remaining clusters, which indicates a tissue-specific expression. FKBP8 was only expressed in cluster 4, while EPHA5 and FKBP8 could be substantially distinguished between cluster 2 and cluster 4. Consequently, it seems that endometriosis tissues with different clinical characteristics could be divided into 4 new molecule subgroups. To our delight, we observed a noticeable variation trend in the expression levels of FKBP8 and IQCG in all 4 subgroups. The expression level of FKBP8 showed a dramatic upward trend from cluster 1 to cluster 4. On the contrary, IQCG manifested a pronounced downward trend from cluster 1 to cluster 4, and distinct alterations were noticed among the 4 clusters. Hence, these sample type exclusive genes might be responsible for the early treatment of endometriosis. Next, the results of RT-qPCR and Western blotting showed that IQCG had a more pronounced difference (*p* < 0.001) between the endometriosis and control groups. Combined with the above analysis, we speculate that IQCG could be utilized to distinguish 4 new molecular subgroups.

In order to discover new valid biomarkers, we further inspected 6 hub ATGs by RT-qPCR and Western blotting in clinical samples and primary ESCs, 4 of which were statistically significant (*p* < 0.05). The genes with the most obvious discrepancy were EZH2 (*p* < 0.001) and RND3 (*p* < 0.001). RND3 belongs to the RND subfamily of the RHO family and has been proven to be an anti-proliferation protein. Previous studies showed that the RND3 was linked with the processes of proliferation, apoptosis, autophagy, cell cycle arrest, migration, and invasion (Paysan et al., 2016). Upregulation of RND3 suppressed the cell proliferation of gastric cancer cells and arrested them in the G0/G1 phase of the cell cycle. Additionally, in gastric cancer, chaperone-mediated autophagy contributed to cell proliferation by targeting RND3 (Zhou et al., 2016). Besides, the genetic deletion of RND3 suppressed cell apoptosis via NF-kB signaling in the brain (Dong et al., 2021). As for EZH2, a subunit of the Polycomb repressive complex 2 (PRC2) catalyzing trimethylation of histone H3 lysine 27 (H3K27), it has been shown to induce epithelial-mesenchymal transition (EMT) in several cancer types. The expression of EZH2 was obviously higher in eutopic and ectopic endometrium in the endometriosis group compared with the control group (Zhang et al., 2017). EZH2 silencing promoted autophagy in endometrial cancer cells. Meanwhile, by activating the mTOR signaling, its overexpression reduced the autophagy and survival of trophoblasts in the trophoblastic disease (Wang et al., 2018; Chu et al., 2020). Furthermore, EZH2 has been identified to be a promising tumor biomarker (Cai et al., 2011), and it represents a prognostic biomarker associated with immunosuppression in esophageal squamous carcinoma and hepatocellular carcinoma (Liu et al., 2016; Guo et al., 2020). Next, we calculated the diagnostic value of the 12 hub ATGs for endometriosis using the ROC analysis, and the results showed the high sensitivity and specificity of EZH2 (AUC: 99.0%) and RND3 (AUC: 94.0%) in diagnosing endometriosis. The Spearman correlation analysis results revealed EZH2, IQCG, RCAN1, RND3, PPP2R5E, and EPHA5 were positively associated with Ca125, it is worth mentioning, EZH2 and RND3 especially striking (r > 0 .75, *p* < 0.0001). These findings suggested that EZH2 and RND3 could be novel potential biomarkers for the diagnosis of endometriosis and further confirmed the role of hub ATGs in endometriosis.

In this work, a multifunctional co-expression network was constructed among lncRNA, miRNA, mRNA, and TF to investigate the mechanism of endometriosis. Some of the 12 hub ATGs (RND3, PLK2, AURKA, RCAN1, ppp2R5E, and EZH2) were selected to construct the lncRNA-miRNA-mRNA ceRNA network and lncRNA-TF-mRNA network. A total of 7 differentially expressed miRNAs (hsa-miR-34a-5p, hsa-miR-92b-3p, hsa-miR-27b-3p, hsa-miR-181b-5p, hsa-miR-25-3p, hsa-miR-367-3p, and hsa-miR-181d-5p) and 4 differentially expressed lncRNAs (AC087477.2, AC116667.1, ERICD, and AL645608.1) were overlapped with GSE105765. The genes from the multifunctional network were tightly linked and cross-regulated each other. For example, in the network, MiR-138-5p/EZH2/SIRT1 were involved in the regulation of autophagy. In a cisplatin resistance of ovarian cancer cells study, the upregulation of miR-138-5p enhanced the chemosensitivity of DDP-resistant cells and apoptosis by decreasing the expression of EZH2 (Zhang et al., 2020). Another study demonstrated a binding site of EZH2/SIRT1, and showed EZH2 and SIRT1 also to participate in the regulation of autophagy. Chidamide treatment markedly downregulated histone deacetylase SIRT1, and simultaneously resulted in a dose-dependent upregulation of acetyltransferase hMOF and histone methyltransferase EZH2, which contributed to the autophagy-suppressive role of chidamide (Xu et al., 2020). At present, there are many related studies on the role of EZH2 and SIRT1 in endometriosis. EZH2 expression was significantly elevated in ectopic and eutopic endometrium from women with endometriosis compared with control endometrium (Colón-Caraballo et al., 2015; Brunty et al., 2021); nuclear SIRT1 expression was increased in those with endometriosis and endometriosis-associated ovarian cancer, not in case with ovarian cancer only (Teasley et al., 2020). However, the detailed mechanism of the multifunctional network should be clarified in the future. We believe that our results will contribute to future research endeavors in this direction.

## Conclusion

This work has shown EZH2 and RND3 to have a high value in diagnosing endometriosis and being effective diagnostic biomarkers. Multifactorial networks related to ATGs have been successfully constructed. The four new molecular subgroups of endometriosis presented in this study based on hub ATGs would assist to adapt the intervention in the future. IQCG could be used to independently differentiate among the subgroups. The above-mentioned results would be beneficial to explore the pathogenesis of endometriosis and provide the foundation to detect novel biomarkers and therapeutic targets for endometriosis. In our future work, we plan to perform an in-depth exploration of the action mechanism of ATGs and increase the sample size.

## Data Availability

The original contributions presented in the study are included in the article/Supplementary Material, further inquiries can be directed to the corresponding author.
