# Supplementary material for: Identification and Analysis of Potential Autophagy-Related Biomarkers in Endometriosis by WGCNA
Source: Front Mol Biosci. 2021 Nov 1;8:743012. doi: 10.3389/fmolb.2021.743012 (PMC8591037; doi:10.3389/fmolb.2021.743012)
Supplement: Supplementary file 1 [file DataSheet1.zip › Supplementary Figures and Tables/Supplement Legends.PDF]

Supplementary Material

Supplementary Figure Legends

Supplement Figure 1. GO enrichment analysis of Green module (A-C). GO enrichment analysis of Red module (D-F).

Supplement Figure 2. The expression of 12 hub ATGs were validated in the two external datasets of GSE7305 (A) and GSE135485 (B).

Supplementary Table Legends

Supplement Table 1. 1928 ATGs in dataset GSE51981.

Supplement Table 2. The ATGs in each key module.

Supplement Table 3. Differentially expressed genes in GSE51981.

Supplement Table 4. One-way analyses of variance of FKBP8 and IQCG in four cluster.

Supplement Table 5. Interactions between miRNA and hub ATGs.

Supplement Table 6. Interactions between miRNA and lncRNA.

Supplement Table 7. Interactions between TF and mRNA.
