# Supplementary figures and images for: Identification and Analysis of Potential Autophagy-Related Biomarkers in Endometriosis by WGCNA
Source: Front Mol Biosci. 2021 Nov 1;8:743012. doi: 10.3389/fmolb.2021.743012 (PMC8591037; doi:10.3389/fmolb.2021.743012)

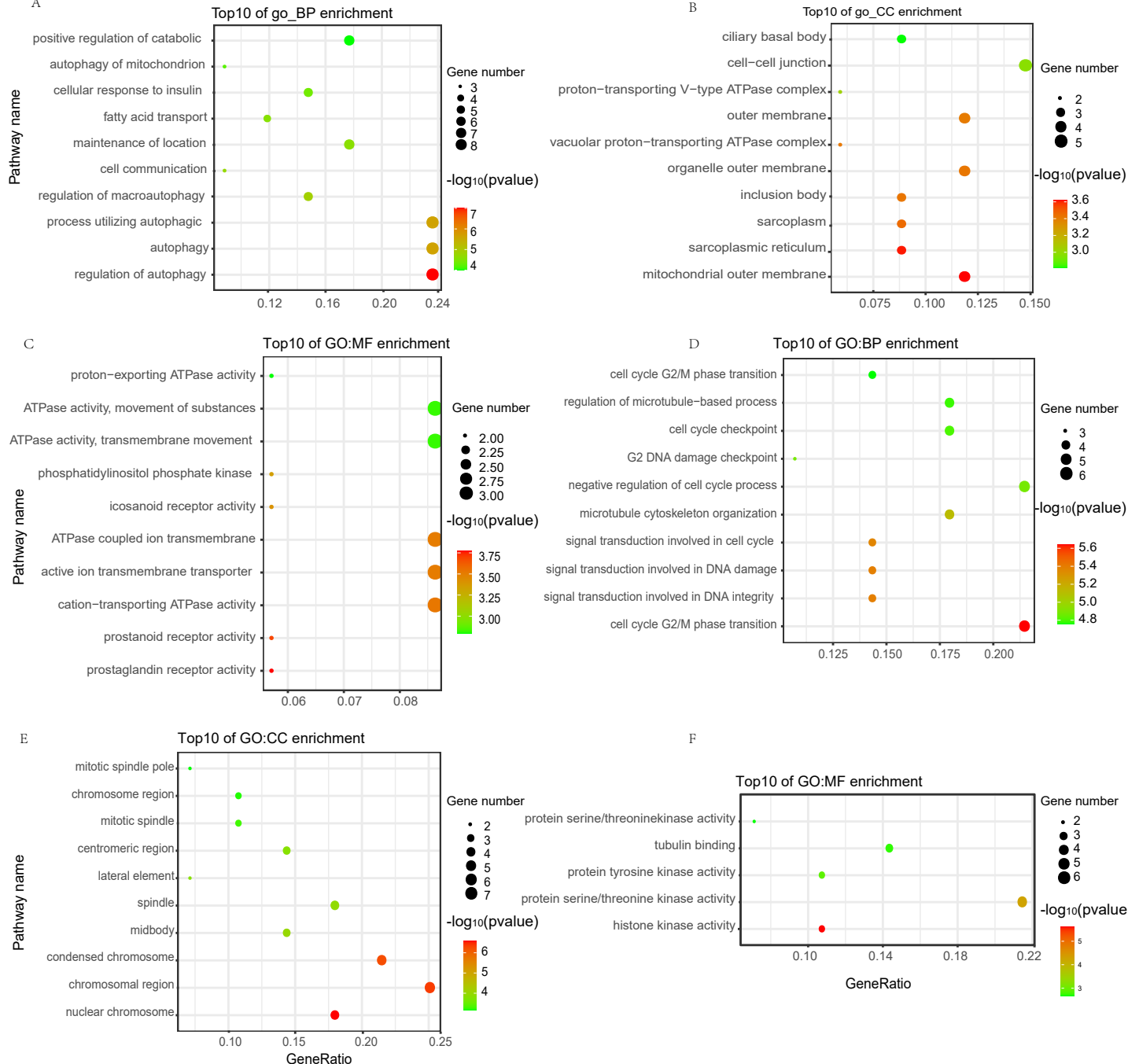

Supplement: Supplementary file 1 [file DataSheet1.zip › Supplementary Figures and Tables/Supplementary Figure 1.pdf]

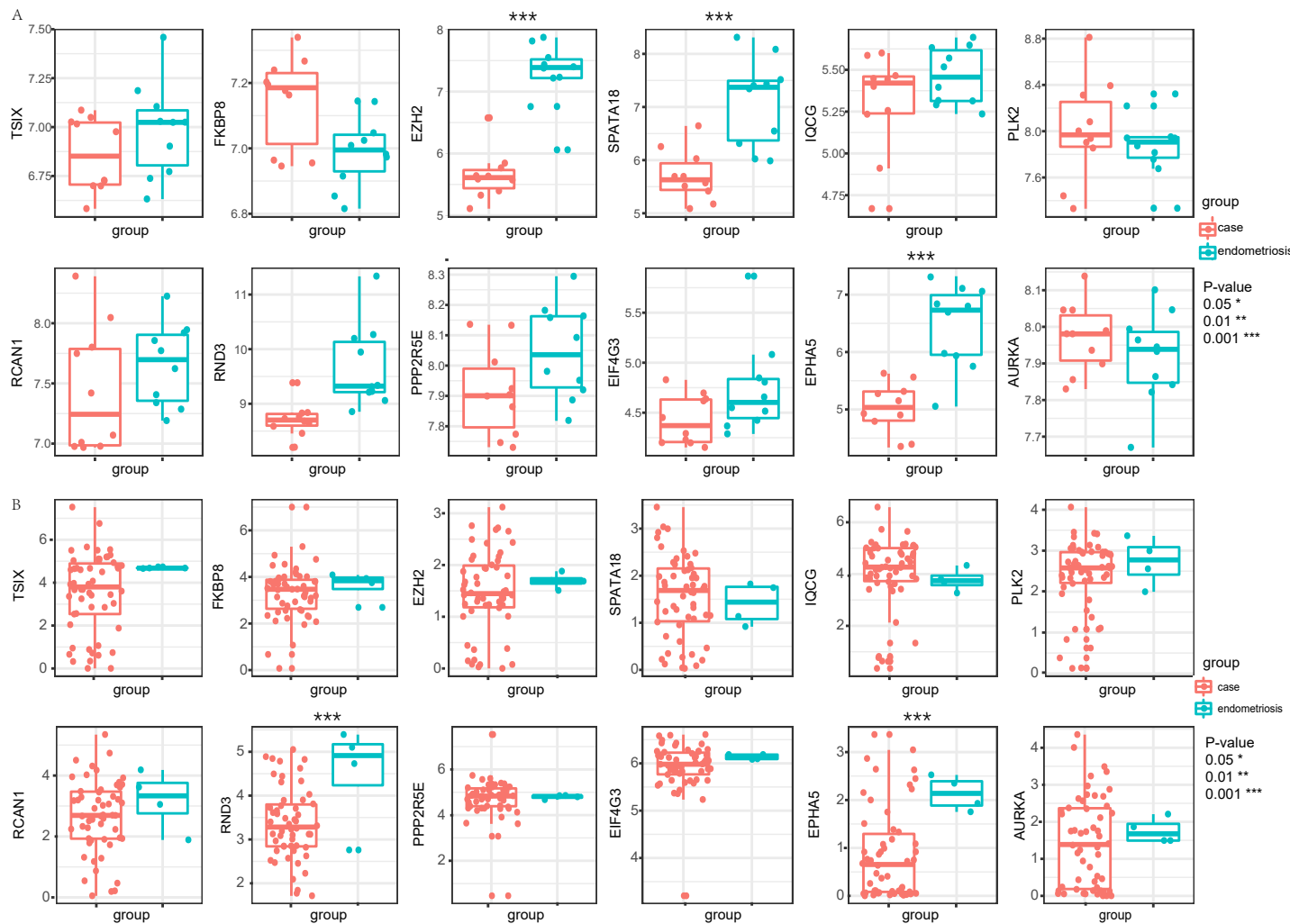

Supplement: Supplementary file 1 [file DataSheet1.zip › Supplementary Figures and Tables/Supplementary Figure 2.pdf]
